# Quality of life of patients with schizophrenia

**DOI:** 10.1192/j.eurpsy.2022.2060

**Published:** 2022-09-01

**Authors:** A. Guermazi, N. Smaoui, D. Jardak, S. Omri, R. Feki, I. Gassara, M. Maalej, N. Charfi, L. Zouari, J. Ben Thabet, M. Maalej

**Affiliations:** Hedi Chaker University Hospital, Sfax, Tunisia, Department Of Psychiatry C, Sfax, Tunisia

**Keywords:** Quality of Life, schizophrénia, 36 item Short-Form Health Survey, Schizoaffective disorder

## Abstract

**Introduction:**

Schizophrenia (SCZ) is considered one of the most disabling mental illnesses with significant family, social and occupational repercussions resulting in impaired quality of life (QOL).

**Objectives:**

To assess the QOL of patients with SCZ or schizoaffective disorder (SAD) and to analyze the sociodemographic and clinical factors associated with its alteration.

**Methods:**

This was a cross-sectional, descriptive and analytical study, which began in December 2019, conducted with 60 subjects followed for SCZ or SAD, at the psychiatry outpatient unit of the Hedi Chaker University Hospital in Sfax (Tunisia). General, clinical and therapeutic data were collected using a pre-established questionnaire. QOL was assessed with the «36 item Short-Form Health Survey» (SF-36).

**Results:**

Patients enrolled had SCZ in 78.2% and SAD in 21.8% of cases. The mean age was 47.2 years and the sex ratio M/F was 4.5. They were single in 63.7% of cases and unemployed in 61.8%. Psychiatric family history, the presence of personal somatic illnesses and tobacco use were found in 43.6%, 61.8% and 67.3% of cases, respectively. The average QOL score was 57.7, the average physical health score was 61.1, and the average mental health score was 54.3. Female sex (p=0.02), being single (p=0.039), lack of work activity (p=0.00), tobacco use (p=0.05), and presence of medical history (p=0.034) were statistically correlated with impaired QOL.

**Conclusions:**

QOL in SCZ and SAD is impaired. This result encourages us to conceive of the patient in his whole life and not only from the point of view of the disease.

**Disclosure:**

No significant relationships.

